# The Stimulating Effect of Rosmarinic Acid and Extracts from Rosemary and Lemon Balm on Collagen Type I Biosynthesis in Osteogenesis Imperfecta Type I Skin Fibroblasts

**DOI:** 10.3390/pharmaceutics13070938

**Published:** 2021-06-23

**Authors:** Joanna Sutkowska, Natalia Hupert, Katarzyna Gawron, Jakub W. Strawa, Michał Tomczyk, Antonella Forlino, Anna Galicka

**Affiliations:** 1 Department of Medical Chemistry, Medical University of Bialystok, ul. Mickiewicza 2A, 15-222 Bialystok, Poland; joanna.sutkowska@umb.edu.pl; 2 Department of Molecular Biology and Genetics, Faculty of Medical Sciences in Katowice, Medical University of Silesia, 40-752 Katowice, Poland; natalia.hupert@sum.edu.pl (N.H.); kgawron@sum.edu.pl (K.G.); 3 Department of Pharmacognosy, Medical University of Bialystok, ul. Mickiewicza 2A, 15-230 Bialystok, Poland; jakub.strawa@umb.edu.pl (J.W.S.); michal.tomczyk@umb.edu.pl (M.T.); 4 Department of Molecular Medicine, Biochemistry Unit, University of Pavia, 27100 Pavia, Italy; aforlino@unipv.it

**Keywords:** rosemary extract, lemon balm extract, rosmarinic acid, collagen type I, skin fibroblasts, osteogenesis imperfecta type I

## Abstract

Rosemary extract (RE) and lemon balm extract (LBE) attract particular attention of pharmacists due to their high therapeutic potential. Osteogenesis imperfecta (OI) type I is a heritable disease caused by mutations in type I collagen and characterized by its reduced amount. The aim of the study was to evaluate the effect of the extracts and rosmarinic acid (RA) on collagen type I level in OI skin fibroblasts. Phytochemical analysis of RE and LBE was carried out by liquid chromatography–photodiode array detection–mass spectrometry. The expression of collagen type I at transcript and protein levels was analyzed by qPCR, ELISA, SDS-urea PAGE, and Western blot. In OI patient’s fibroblasts the exposure to the extracts (0.1–100 µg/mL) and RA (0.1–100 µM) significantly increased collagen type I and the best results were obtained with 0.1–10 µM RA and 0.1–10 µg/mL of the extracts. LBE showed a greater stimulating effect than RE, likely due to a higher RA content. Moreover, collagen type III expression and matrix metalloproteinase (MMP-1, -2, -9) activity remained unchanged or decreased. The obtained data support the clinical potential of RA-rich extracts and RA itself in modulating the quantitative defect of type I collagen in type I OI.

## 1. Introduction

**Figure 1:**
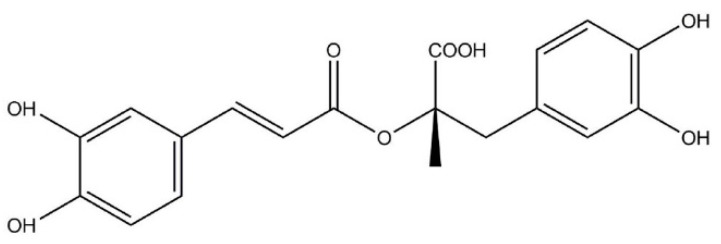
Chemical structure of rosmarinic acid (RA).

Medicinal herbs and herbal remedies have been widely used in traditional medicine. Furthermore, their use is growing rapidly in modern therapy of various ailments and diseases. Rosemary (*Rosmarinus officinalis* L.) and lemon balm (*Melissa officinalis* L.) are perennial plants from the Lamiaceae family, naturally occurring in the Mediterranean Sea and West Asia, as well as being commonly cultivated in Europe and North America [1,2]. They are widely used in the pharmaceutical, food and cosmetic industries due to their various biological activities. which include antibacterial, antifungal, antiviral, antioxidant, anti-inflammatory, immunomodulating, antidepressant, anticoagulant, antihyperglycemic, antinociceptive, antiulcer, and antitumor [1,2,3,4,5]. For the broad effects of these herbs on human health, a whole range of active compounds are responsible that can act alone or in various combinations. An important component of rosemary extracts (RE) and lemon balm extract (LBE) that independently showed a wide range of pharmacological activities and therapeutic applications is rosmarinic acid (RA), an ester of caffeic acid and 3, 4–dihydroxyphenyl lactic acid (Figure 1).

The characteristics of the RA have been discussed extensively in the reviews [6,7,8,9]. Based on the results of in vitro studies and clinical trials, it can be assumed that it brings many benefits in the treatment of disorders related to the nervous, cardiovascular, gastrointestinal, hepatic, endocrine, respiratory and reproductive systems, cancer diseases, osteoarthritis, allergic rhinitis, periodontal disease, acute pancreatitis, and metabolic syndrome.

The antitumor activity of rosemary and lemon balm extracts, well-studied in vitro and in vivo, is associated with an increase in antioxidant enzyme activity, a reduction in tumor-stimulating inflammation, suppression of tumor angiogenesis, regulation of epigenetic modifications, modulation of immune responses, alteration of hormone signaling, modification of specific metabolic pathways, stimulation of cancer suppressive genes, and cell death programming [3,4,5,10]. Rosemary and its derivatives can also inhibit the metastasis process and enhance the effect of chemotherapy by reducing the phenomenon of chemoresistance [10].

There are also reports of a beneficial effect of the RA and extracts (RE and LBE) on the skin with particular interest in the field of cosmetology and medicine in supporting the treatment of skin diseases [11,12,13,14,15,16,17,18,19]. RA has shown its efficacy in treating allergic disorders in clinical trials [11]. Lee et al. [12] in an in vivo study revealed its possible use as a therapeutic agent in atopic dermatitis, one of the inflammatory disorders of the skin. Research on the beneficial effects of RE on the skin, including the protective effect against ultraviolet (UV) radiation and melanoma, reduction of skin damage, anti-aging effect by increasing skin hydration and elasticity, and accelerated wound healing, was described in the review by de Macedo et al. [13]. The mechanisms of the anti-aging action of this extract include the reduction of reactive oxygen species level and expression of p53 protein, prevention of DNA damage, down-regulation of matrix metalloproteinases (MMP-1 and MMP-3) mRNA as well as inhibition of lipid peroxidation [14,15,16]. Rosemary has been shown to be effective as a preservative in both food and cosmetics, often used in combination with other extracts [15,16]. Rosemary leaf extract is used as skin conditioner and is present up to 10% in hand and body products and 3% in preparations for eye shadows, soaps and detergents [15]. It also prevents herpes infections, dermatomycosis, cellulitis, and pain [15].

LBE also showed many beneficial effects on the skin, including the protective effect against skin damage caused by UVB radiation [17], improving skin elasticity in healthy adults [18] or treatment of acne, and infectious diseases of the skin [19].

Taking into account a number of extremely valuable health-promoting properties of RA and extracts and relying on our previous research on the stimulating effect of polyphenols on type I collagen in human skin fibroblast [20,21,22], we assumed that they could have a positive impact on the quantitative collagen type I defect in osteogenesis imperfecta (OI) type I. OI is a phenotypically and genetically heterogeneous rare disease of connective tissue originally classified into four types: mild type I, lethal type II, progressive-deforming type III, and moderately mild type IV [23]. Most (85–90%) patients experience qualitative or quantitative abnormalities of type I collagen, due to a mutation in one of two genes *COL1A1*/*COL1A2* encoding this protein [24,25,26,27]. Currently, the classification has expanded to 20 types due to the discovery of many other causative genes related more or less closely to collagen type I biosynthesis, modification, secretion or processing [25,26,27]. Although OI is primarily a bone disease, extra-skeletal defects are described in several patients [25,26,27,28,29]. Abnormalities of collagen type I may manifest as excessive mobility of ligaments, skin fragility, muscle weakness, hearing loss, and dentinogenesis imperfecta. Most of the research on the mechanisms of the pathophysiology of this disease is carried out on patients’ skin fibroblasts because, compared to osteoblasts, they are more accessible and easier to culture. Collagen is the main component of the skin extracellular matrix (ECM) as it constitutes 75% of the dry weight of the skin, and type I collagen accounts for 80 to 90% of the total collagens [30,31]. Due to the significant role of this protein in the skin, it acts as a scaffold and is responsible for mechanical strength; decreasing collagen biosynthesis by approximately 50% in OI type I may be associated with impairment of mechanical properties of the tissue disrupting its proper structure and function [28,29]. Therefore, increasing biosynthesis of collagen type I could at least partially restore the proper composition of the skin ECM and improve its properties in this disease and likely have beneficial effects on bone as well, considering the high amount of collagen type I in this tissue. In addition to demonstrating the beneficial effect of natural compounds on the biosynthesis of collagen type I in skin fibroblasts of a patient with OI type I, their influence on the activity of MMPs involved in collagen and skin homeostasis, such as MMP-1, MMP-2, and MMP-9, was also investigated.

## 2. Materials and Methods

### 2.1. Chemicals

Acetonitrile Optima was purchased from Fisher Chemical (Thermo Fisher Scientific, Leicestershire, UK), ultra-pure water (UPW) (resistivity of 18.2 MΩ-cm was obtained using the POLWATER DL3-100 system (Labopol, Kraków, Poland). Formic acid (FA) (Ph. Eur., Merck, Darmstadt, Germany) was used as the mobile phase modifier. Ethanol used to extraction was purchased from POCH Basic (POCH, Gliwice, Poland). Hesperidin and rosmarinic acid were purchased from CAYMAN (Ann Arbor, Michigan, USA). Apigenin (purity > 96%) was isolated from *Lychnis flos-cuculi* herb [32], luteolin 7-*O*-glucoside (purity > 96%) was isolated from *Bidens cernua* [33], hispidulin 7-*O*-glucoside (purity > 96%) was isolated from flowers of *Cirsium rivulare* [34], hispidulin (purity > 96%) was isolated from flowers of *C. rivulare* [35], cirsimaritin (purity > 96%) was isolated from leaf buds of white birch [36]. Dulbecco’s minimal essential medium (DMEM), fetal bovine serum (FBS) and phosphate-buffered saline (PBS) were purchased from Gibco (Thermo Fisher Scientific, Waltham, MA, USA). Penicillin, streptomycin, and glutamine were obtained from Quality Biologicals Inc. (Gaithersburg, MD, USA). Radioimmunoprecipitation assay (RIPA) buffer, protease inhibitor cocktail (P8340), dimethyl sulfoxide (DMSO), 4-aminophenylmercuric acetate (APMA), magnesium L-ascorbate, and pepsin were provided by Sigma–Aldrich Corp. (St. Louis, MO, USA).

### 2.2. Preparation of RE and LBE Extracts

Rosemary (*R. officinalis*) and lemon balm (*M. officinalis*) leaves were collected from the garden of medicinal plants from the Medical University of Białystok, Poland in July 2018. Samples of the collected plant material were identified based on the scientific botanical literature and its morphological features by one of the authors (M.T.). Immediately after harvesting, the plant material was dried in the dark and well-ventilated room. Dry, powdered plant materials (each 5.0 g) were sonificated (Sonic-5, Polsonic, Warsaw, Poland) at 40 °C in 50% (*v*/*v*) ethanolic solution (5.0 g sample/50 mL) for 30 min, twice. Filtered supernatants were reduced to dryness under vacuum (Büchi System, Flawil, Switzerland) at a controlled temperature (40 ± 2 °C), further suspended in water, and freeze-dried using a vacuum concentrator (Labconco, Kansas City, MO, USA) until constant weight. With this procedure, the observed yield values were 1.18 g for RE (yield 23.65%), and 1.25 g for LBE (yield 22.5%).

### 2.3. Liquid Chromatography-Photo-Diode Array Detector-Mass Spectrometry (LC-PDA-MS) Analysis of RE and LBE

The assessment of chemical composition of each extract (RE, LBE) was carried out on a 1260 Infinity chromatograph (Agilent, Santa Clara, CA, USA) consisting of binary pump, a column oven, and photo-diode array (PDA) detector over 55 min period. The separation was performed using a Kinetex XB C18 column (150 × 3 mm, 2.6 µm) (Phenomenex, Torrance, CA, USA). The mobile phase was 0.1% (*v*/*v*) FA in water (A) and 0.1% FA in acetonitrile (B). The separation was achieved by a gradient of 0–2 min 1% B; 2–20 min 1–20% B; 20–40 min 20–75% B; 40–45 min 75–95% B; 45–48 min 95% B; 48–49 min 95–1% B; 49–55 min 1% B. The flow rate was 0.5 mL/min and the column temperature was maintained at 25 ± 0.8 °C. The UV–Vis spectra was recorded from 190 to 540 nm with selective wavelength monitoring at 280 nm. Mass spectrometry (MS) detection was carried out on a 6230 MS/TOF mass spectrometer (Agilent, Santa Clara, CA, USA) equipped with an electrospray ionization source with Agilent Jet Stream thermal focusing. The parameters used for ionization source were set as follows: drying and sheath gas flow: 12 L/min; nebulizer: 35 psi; source temperature 350 °C; ion spray voltage 4500 V for the positive mode analysis. The data were collected in 115–1900 *m*/*z* range and processing was performed using Mass Hunter qualitative analysis software.

### 2.4. Preparation of Standard Solution for Quantification

Standard solutions with concentrations of 0.1; 0.3; 0.5; 0.8; 1.0, 3.0 mg/mL were prepared from the stock standard solution by dilution method and were immediately used to determine the standard curve.

### 2.5. Method Validation

#### 2.5.1. Selectivity

Three solutions were made for both tested extracts to exclude the occurrence of co-elution. Analysis with the use of PDA detector at three selected wavelengths and additional MS detection were used. The presence of other compounds during the retention of the substance being determined was excluded.

#### 2.5.2. Linearity

Standard solutions of RA at 6 concentration levels were analyzed in triplicate. Calibration curves were generated using linear regression on the plots of peak area of each standard versus amount injected to column. Linear regression parameters for the standard curve were determined using ANOVA. Statistical significance has been confirmed. Calculations were made using MS Excel 2016.

#### 2.5.3. Limits of Detection (LOD) and Quantification (LOQ)

In accordance with the International Council for Harmonisation of Technical Requirements for Pharmaceuticals for Human Use (ICH) recommendations [37] for the standard used, the detection limit and limit of quantification have been set as equations 3 SD/a and 10 SD/a, respectively. The slope of the calibration curve was taken as standard deviation as the product of the standard error of the intercept and square root number of repetitions (see Table 1).

#### 2.5.4. Accuracy and Precision

Accuracy was determined as the theoretical recovery using detector response and regression equation parameters. Precision was determined using six concentration levels analyzed in triplicate and expressed as a coefficient of variation (%CV) (see Table 1).

### 2.6. Primary Human Skin Fibroblasts

The study was performed on the primary skin fibroblast line obtained from an OI type I patient carrying an out of frame deletion in exon 5 of the *COL1A1* gene (g.2674 del T, c.459delT, p.Gly145Alafs * 111, unpublished data) in accordance with the Declaration of Helsinki and was approved by the Bioethical Committee of the Jagiellonian University in Kraków, Poland (KBET/108/B/2007, 31 May 2011). Normal human skin fibroblast line CCD25Sk was purchased from the American Type Culture Collection and used as control. Fibroblasts were used between passages 2–6 and cultured in Dulbecco’s minimal essential medium (DMEM) supplemented with 10% fetal bovine serum (FBS), 2 mM glutamine, 50 U/mL penicillin and 50 µg/mL streptomycin in an incubator at 37 °C and humidified atmosphere containing 5% CO_2_. For the experiments, cells were grown to 90% confluence and 2 h before treatment, culture medium was replaced with fresh serum-free DMEM supplemented with magnesium ascorbate (25 µg/mL). Compounds were dissolved in dimethyl sulfoxide (DMSO) and stored at 4 °C as the concentrated stock solutions. Fresh dilutions in DMEM were made prior to adding them to cell cultures for the final concentrations: RA (0.1–100 µM), RE (0.1–100 µg/mL) and LBE (0.1–100 µg/mL), with DMSO not exceeding 0.1%.

### 2.7. Cell Viability Assay

The assay was performed using [(4,5-dimethylthiazol-2-yl)-2,5-diphenyltetrazolium bromide] (MTT). After 24 h treatment of cells with the compounds at 37 °C, the culture medium was removed from the wells, and cells were washed three times with PBS. Then 1 mL of MTT solution (0.5 mg/mL) was added to each well and incubated for 4 h at the same temperature. After this time MTT solution was replaced with 1 mL of 0.1 M HCl in absolute isopropanol and subjected to thoroughly shaking to dissolve the resulting formazan crystals. Cell viability was evaluated by the measurements of the absorbance (570 nm) using a microplate reader (TECAN, Männedorf, Switzerland).

### 2.8. Quantitative Real-Time PCR Analysis

Total RNA of cells was isolated using a Total RNA Mini Plus concentrator (A&A Biotechnology, Gdynia, Poland) according to the manufacturer’s instructions. The concentration of obtained RNA was measured using the NanoDrop 2000 spectrophotometer (Thermo Fisher Scientific, Waltham, MA, USA). Equal amounts of RNA (1 µg) were subjected to the synthesis of complementary DNA (cDNA) with the use of the cDNA Synthesis Kit (Bioline, London, UK). Each cDNA was diluted 10-fold and used as a template for quantitative real-time PCR (qRT-PCR) assay using SensiFAST™ SYBR Kit (Bioline, London, UK). The qRT-PCR was performed in CFX96 real-time system thermal cycler (Bio-Rad, Hercules, CA, USA) in the final volume of 10 μL of the reaction mixture. The sequences of primers (Genomed, Warsaw, Poland) used to analyze gene expression were as follows: *COL1A1*, forward 5′-ATG TCT AGG GTC TAG ACA TGT TCA-3′ and reverse 5′-CCT TGC CGT TGT CGC AGA CG-3′; glyceraldehyde-3-phosphate dehydrogenase *(GAPDH)*, forward 5′-CTC TGC TCC TCC TGT TCG AC-3′ and reverse 5′-GCC CAA TAC GAC CAA ATC C-3′. Three replicates in double repeats were conducted for each reaction. The qRT-PCR proceeded according to the following program: 30 s at 95 °C for initial denaturation, followed by 40 cycles (10 s at 95 °C, 10 s at 60 °C and 20 s at 72 °C). The specificity of products of each amplification was verified by the analysis of the melting curves. The relative gene expression level was calculated using the 2^−ΔΔCT^ method in the CFX96 real-time PCR system.

### 2.9. Enzyme-Linked Immunosorbent Assay (ELISA) Measurement for Procollagen Type I

The amount of procollagen type I in cell lysates and secreted by fibroblasts was measured using Human pro-Collagen I alpha 1 Simple Step Elisa Kit (Abcam, Cambridge, UK). After 24 h treatment of cells with tested compounds, the experimental media were collected and centrifuged at 2000× *g* for 10 min. The concentration of protein in cell culture supernatants was determined with Coomassie Plus—The Better Bradford Assay Reagent (Thermo Fisher Scientific, Rockford, IL, USA). Cells were washed three times with PBS and harvested with Extraction buffer provided with the assay. After incubation on ice for 20 min samples were centrifuged at 18,000× *g* for 20 min at 4 °C. The concentration of protein in cell lysates was determined using BCA Protein Assay Kit (Pierce, Rockford, IL, USA). A standard curve was prepared using Procollagen I alpha 1 standard in the concentration of 10–1000 pg/mL. Aliquots of samples (50 µL/well) of an appropriately diluted cell lysates and experimental media, containing 0.5–1 mg of total proteins, were added to a 96-well microtitre plate coated with procollagen type I standard and then the manufacturer’s protocol was followed. The assays were done in duplicates in three independent experiments. The secretion of procollagen was calculated by dividing the amount of procollagen released into the cultured medium by the sum of medium and intracellular collagen.

### 2.10. Western Blot

The cultured media collected after experiments were concentrated 10 times, cells were harvested in RIPA buffer (Sigma–Aldrich Corp., St. Louis, MO, USA) supplemented with protease inhibitor cocktail (P8340) (Sigma–Aldrich Corp., St. Louis, MO, USA). An equal amount of protein (20 µg) was loaded on 7.5% polyacrylamide gel and after electrophoresis proteins were transferred onto Immobilon-P Transfer membrane (Merck Millipore Ltd., Tullagreen, Carrigtwohill, County Cork, Ireland). Then membrane was washed in TBS-T (50 mM Tris-HCl, 500 mM NaCl, pH 7.5 containing 0.05% (*v*/*v*) Tween 20) and blocked with 5% (*w*/*v*) non-fat dried milk in TBS-T at room temperature for 1 h. After this time, the membrane was washed three times with TBS-T and incubated overnight at 4 °C with primary antibodies: mouse anti-collagen type I (1:1000; Santa Cruz Biotechnology Inc., Santa Cruz, CA, USA) or rabbit anti- β-actin (1:1000; Sigma-Aldrich Corp., St. Louis, MO, USA) as a loading control. The appropriate secondary anti-mouse immunoglobulin G (IgG) (Sigma–Aldrich Corp., St. Louis, MO, USA) and anti-rabbit horseradish peroxidase conjugated antibodies (Cell Signaling Technology, Massachusetts, USA) at dilution of 1:2000 were added and membrane was incubated for 1 h at room temperature with slow mixing. After washing (3 times for 10 min with TBS-T) membrane was developed using a Westar Supernova Chemiluminescent Substrate for Western Blotting (Cyanagen, Bologna, Italy) and documented with the use of BioSpectrum Imaging System gel documentation apparatus (UVP, Upland, CA, USA). The intensity of the protein bands was measured by densitometry, for which an imaging densitometer (G: BOX, Syngene, Cambridge, UK) was used.

### 2.11. Steady-State Analysis of Type I Collagen

Confluent fibroblast cultures were incubated for 2 h in serum-free medium containing magnesium ascorbate (25 µg/mL), and after the adding of RA, RE, and LBE incubation was continued for 16 h. Procollagens were harvested from the media and cell layer and precipitated overnight at 4 °C with ammonium sulfate (176 mg/mL). After centrifugation at 37,000× *g*, pellets were dissolved in 0.5 M CH_3_COOH. To obtain collagen, procollagen solution was digested with pepsin (50 μg/mL) for 4 h at 4 °C. Procollagen chains and collagen chains were separated on 5% SDS-urea-polyacrylamide gels under reducing and non-reducing conditions, respectively, and visualized by silver staining [38]. The intensity of collagen bands was measured by densitometry (G: BOX, Syngene, Cambridge, UK).

### 2.12. Zymography

The fibroblast conditioned medium containing an equal amount of total proteins was activated with 1 mM APMA, mixed with four times concentrated sample buffer and load on 10% SDS-polyacrylamide gel containing 1 mg/mL gelatin (Sigma–Aldrich Corp., St. Louis, MO, USA) for detection of MMP-1, MMP-2, and MMP-9 activities, respectively. After electrophoresis, the gels were transferred to a 2.5% Triton X-100 solution to remove SDS. Then the gels were placed in the incubation buffer (50 mM Tris-HCl, pH 8.0, supplemented with 5 mM CaCl_2_, 5 µM ZnCl_2_ and 0.02% NaN_3_) overnight at 37 °C. For gel staining 0.5% Coomassie Brilliant Blue R-250 in solution containing 40% methanol and 10% acetic acid was used. Finally, they were destained until MMP as transparent stripes on a blue background were visible. Images of the zymograms were subjected to the densitometry (G:BOX, Syngene, Cambridge, UK).

### 2.13. Statistical Analysis

The results were statistically analyzed using the Statistica 12 software (StatSoft, Tulsa, OK, USA). They were presented as the mean ± standard deviation (SD). Statistical differences were estimated using a one-way ANOVA followed by Tukey’s test. Values of *p* < 0.05 were considered to indicate statistically significant differences.

## 3. Results

### 3.1. Qualitative and Quantitative Analysis of RE and LBE

**Figure 2:**
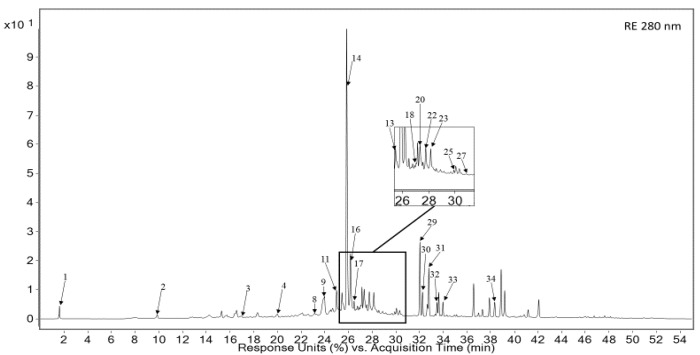
UV spectrum of major constituents of rosemary extract (280 nm).

**Figure 3:**
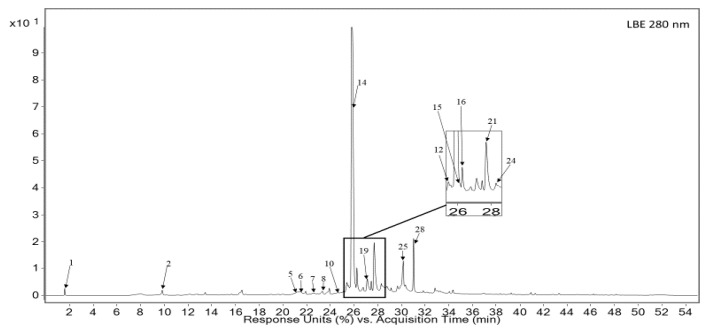
UV spectrum of major constituents of lemon balm extract (280 nm).

Detailed phytochemical analysis of the secondary metabolites present in both tested extracts (RE and LBE) showed the presence of 34 different compounds. The fingerprints of the analyzed extracts were established using LC-PDA-MS method. The analysis revealed constituents comprising polyphenols as caffeic acid derivatives (14, 24), flavonoids such as luteolin (7, 8, 16, 20, 22, 23) and apigenin (10, 25) derivatives, respectively, and related compounds such as diterpenes (29, 31, 32). The LC-MS analysis is summarized in Table 2, Figure 2, Figure 3. Finally, we determined the quantitative content of the dominant RA component in both tested extracts. Quantitative analysis of its content showed its high concentration of 27.23 ± 0.54 mg/g and 80.26 ± 1.24 mg/g for RE and LBE, respectively (Table 3).

### 3.2. The Influence of RA, RE and LBE on the Viabiliy of Normal and OI Fibroblasts

**Figure 4:**
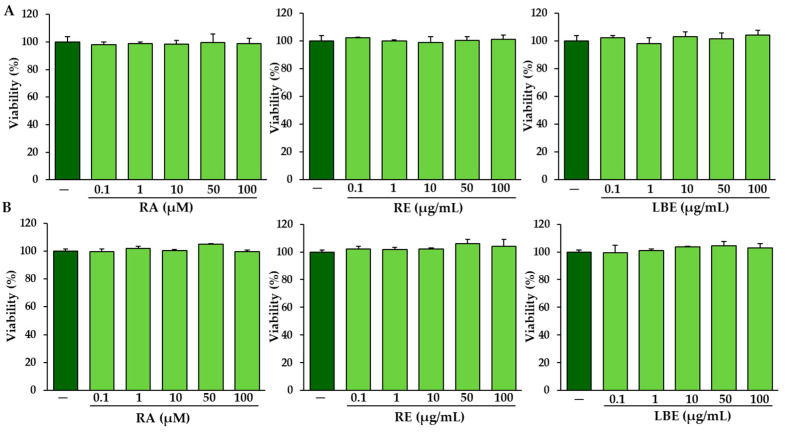
Effects of rosmarinic acid (RA) at concentrations of 0.1–100 µM, and rosemary extract (RE) and lemon balm extract (LBE) at concentrations of 0.1–100 µg/mL each on the viability of normal (**A**) and OI (**B**) skin fibroblasts. Values represent the means of percentage of the untreated cells ± SD (*n* = 5).

After 24 h treatment of normal and OI skin fibroblasts with RA at concentrations of 0.1–100 µM and with extracts (RE and LBE) at concentrations of 0.1–100 µg/mL each, no significant effect on cell viability was found (Figure 4).

### 3.3. Effect of RA, RE and LBE on the Content of Intracellular and Secreted Type I Procollagen in OI Fibroblasts Determined by ELISA

**Figure 5:**
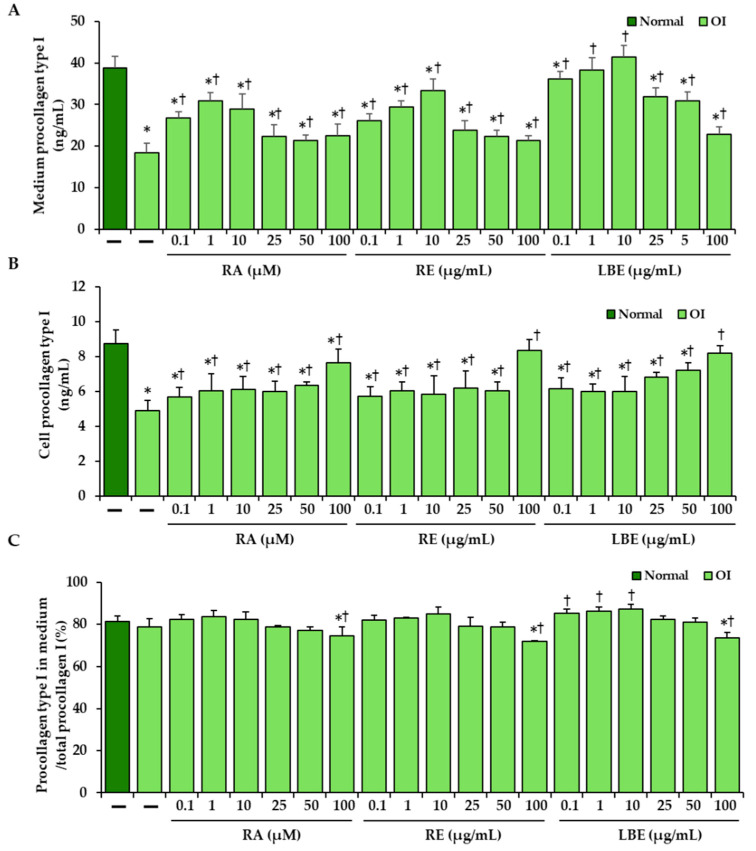
The influence of rosmarinic acid (RA), rosemary extract (RE), and lemon balm extract (LBE) on the amount of secreted (**A**) and intracellularly retained (**B**) type I procollagen in OI cells. (**C**) Secretion expressed as the ratio of secreted type I procollagen to total procollagen I. Values represent the mean ± SD of three experiments done in duplicate. * *p* < 0.05, OI cells vs. normal cells; † *p* < 0.05, OI treated cells vs. OI untreated cells.

Human pro-Collagen I alpha 1 Simple Step Elisa Kit (Abcam, Cambridge, UK) was used to estimate the effect of RA and extracts (RE and LBE) on the amount of intracellular and secreted type I procollagen. In OI skin fibroblasts, the amount of intracellular and secreted type I procollagen was reduced by about half compared to normal cells, while the exposure of OI cells to RA at concentrations of 0.1–100 µM, to RE at concentrations of 0.1–100 µg/mL, and to LBE at concentrations of 0.1–100 µg/mL resulted in a significant increase in the secreted procollagen at all concentrations (Figure 5A). The highest increase as compared to untreated OI cells was observed in the presence of RA at concentrations of 0.1, 1, and 10 µM and extracts (RE and LBE) at concentrations of 0.1, 1, and 10 µg/mL each. Moreover, LBE at concentrations of 1 and 10 µg/mL normalized the amount of procollagen type I to the level of this protein in the cultured medium of normal cells. In turn, cells treated with RA and extracts also showed an increase in the content of type I procollagen, except for their highest concentrations (100 µM RA, 100 µg/mL RE, 100 µg/mL LBE), to a lesser extent than in the medium (Figure 5B). Secreted procollagen type I accounted for 82.5% and 79.1% of total collagen in normal and OI cells, respectively, while under the influence of 100 µM RA, 100 µg/mL RE, and 100 µg/mL LBE, it decreased to 74.5%, 71.8%, and 73.6%, respectively. In the presence of LBE at concentrations of 0.1, 1, and 10 µg/mL there was the increase in secretion of type I procollagen in OI cells comparable to that showed in the normal cell (Figure 5C).

For subsequent analyzes, we chose the concentrations of RA (0.1, 1, and 10 µM), RE (0.1, 1, and 10 µg/mL), and LBE (0.1, 1, and 10 µg/mL), at which they had the most beneficial effect on procollagen type I in OI fibroblasts.

### 3.4. Analysis of Procollagen and Collagen Type I and III in OI Fibroblasts Treated with RA, RE and LBE

**Figure 6:**
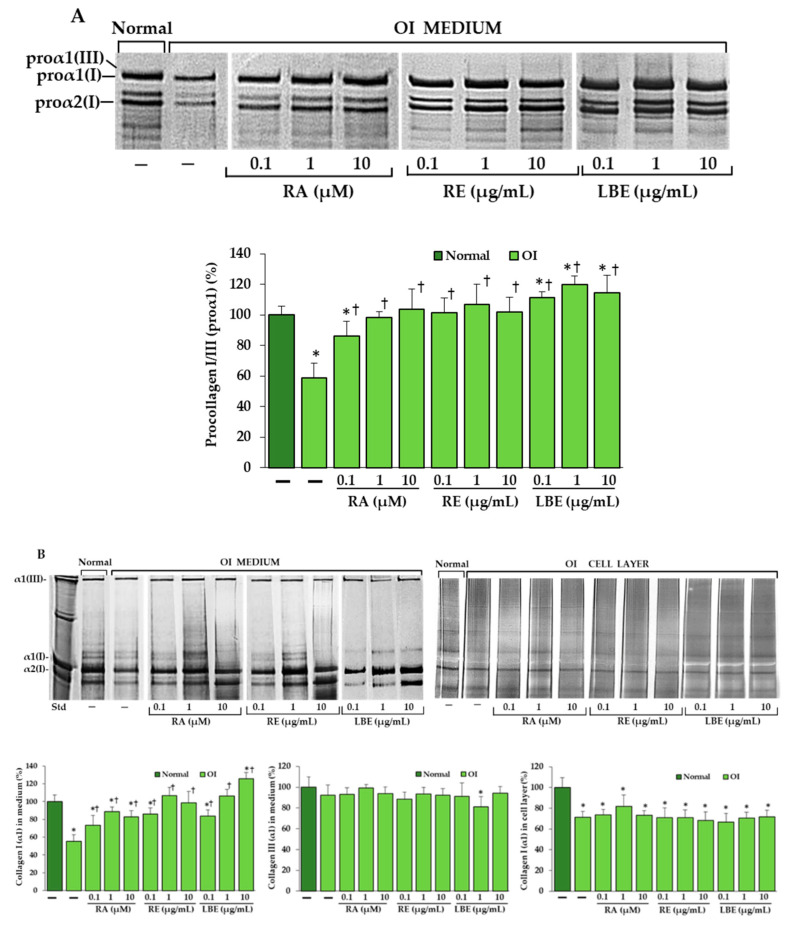
SDS-urea polyacrylamide gel electrophoresis (SDS-urea PAGE) of procollagen type I and III in reducing conditions (**A**) and collagen type I and III in non-reducing conditions (**B**) in OI fibroblasts treated with rosmarinic acid (RA), rosemary extract (RE), and lemon balm extract (LBE); Std—bovine collagen type I (Biocolor Life Science, U.K). Densitometry results represent the mean of three independent experiments; * *p* < 0.05, OI cells vs. normal cells; † *p* < 0.05, OI treated cells vs. OI untreated cells. The data are expressed as a percentage of the control sample assumed as 100%.

SDS-urea polyacrylamide gel electrophoresis (SDS-urea PAGE) was used for the identification and comparison of procollagen and collagen type I and III in treated OI cells. Procollagen type III migrated close to the proα1 of procollagen type I and their levels in OI cultured medium were significantly lower compared to the normal, while an increase is evident after the treatment of OI cells with RA at concentrations of 0.1, 1, 10 µM, and extracts (RE and LBE) at concentrations of 0.1, 1, and 10 µg/mL each (Figure 6A). The increase in the level of type I collagen in the treated OI cells was confirmed after separation of pepsin-digested procollagen under non-reducing conditions, whereas no substantial impact of RA and both extracts (except a slight reduction by 1 µg/mL LBE) on type III collagen was found (Figure 6B). The intensity of the α1(I) bands in cell layer was comparable in the treated and untreated OI cells or slightly increased at 1 µM RA.

### 3.5. Western Blot Analysis of Expression of Collagen Type I in OI Fibroblasts

**Figure 7:**
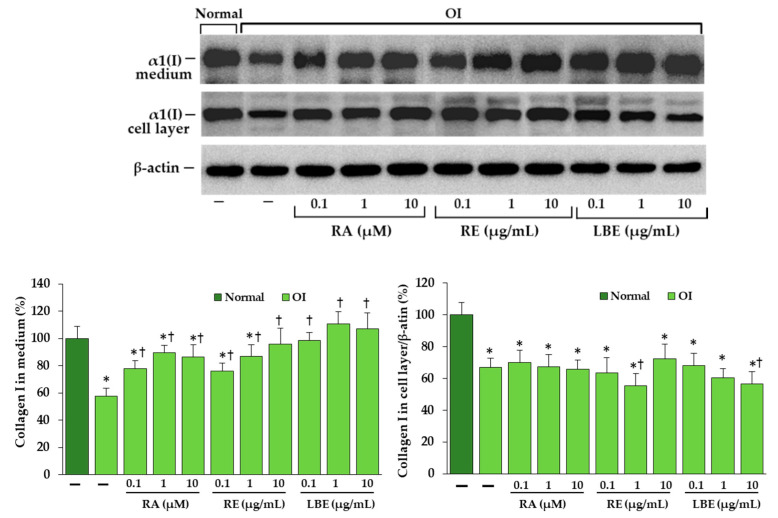
The influence of rosmarinic acid (RA), rosemary extract (RE), and lemon balm extract (LBE) on secreted (in medium) and intracellular type I collagen, β-actin was shown as cell protein loading control. Densitometry results represent the mean of three independent experiments; * *p* < 0.05, OI cells vs. normal cells; † *p* < 0.05, OI treated cells vs. OI untreated cells. The data are expressed as a percentage of the control sample assumed as 100%.

Western blot analysis clearly showed the increase in the secreted type I collagen under the influence of RA and both extracts in OI cells (Figure 7). Densitometric measurements showed the greatest increase, corresponding to the level of collagen secreted by normal cells, in the presence of LBE at concentrations of 0.1, 1 and 10 µg/mL and 10 µg/mL RE. Normalization of intracellular type I collagen to the β-actin showed either no difference in treated versus untreated OI cells or a reduction by RE (1 µg/mL) and LBE (10 µg/mL).

### 3.6. Effect of RA, RE and LBE on the Expression of Collagen Type I at mRNA Level in OI Fibroblasts

**Figure 8:**
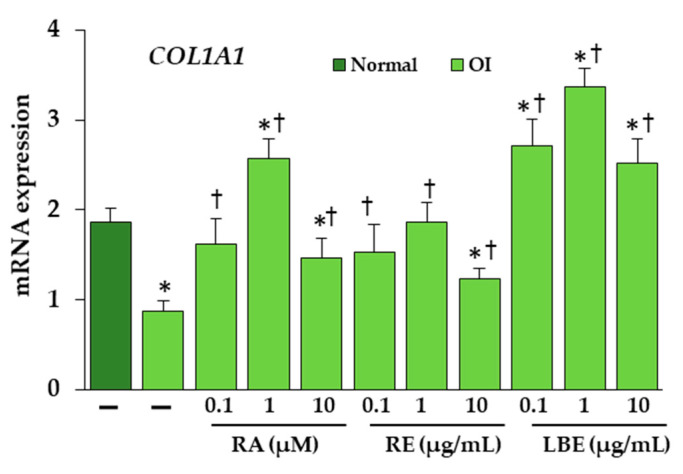
The influence of rosmarinic acid (RA), rosemary extract (RE), and lemon balm extract (LBE) on the expression of *COL1A1* gene encoding α1 of collagen type I in OI skin fibroblasts. Values represent the mean ± SD of three experiments done in duplicate. * *p* < 0.05, OI cells vs. normal cells; † *p* < 0.05, OI treated cells vs. OI untreated cells.

To examine whether the extracts and RA affect collagen type I at mRNA level, the quantitative real-time PCR was performed. As shown in Figure 8, in OI untreated cells, the expression of *COL1A1* gene encoding α1(I) was reduced by about half, while the significant increase was seen in cells treated with RA and extracts. At 0.1 µM RA and 0.1, 1 µg/mL RE, the transcript level was comparable to normal cells, while in the presence of 1 µM RA and 0.1, 1, and 10 µg/mL LBE, *COL1A1* expression was significantly exceeded compared to untreated OI and normal cells.

### 3.7. Effect of RA, RE and LBE on the Activity of MMP-1, MMP-2 and MMP-9 in OI Fibroblasts

**Figure 9:**
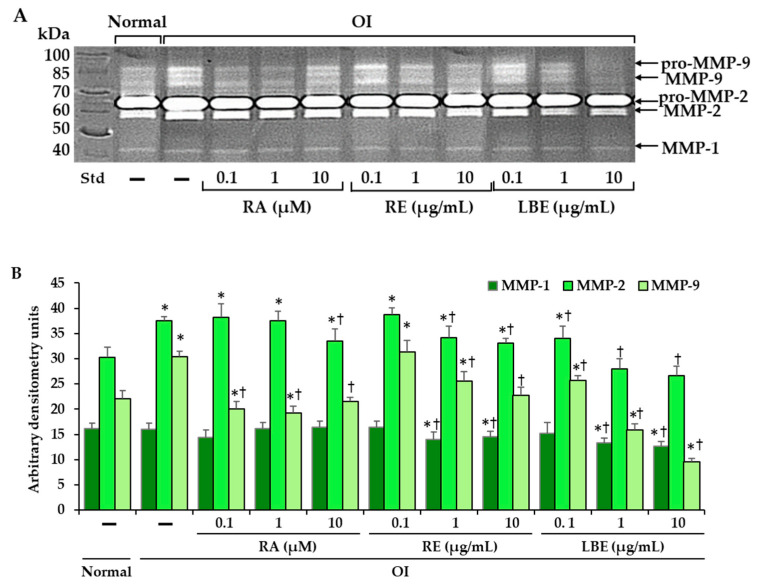
The influence of rosmarinic acid (RA), rosemary extract (RE), and lemon balm extract (LBE) on the activity of MMP-1, MMP-2, and MMP-9 in OI cells determined by zymography (**A**); Std—PageRuler™ Unstained Protein Ladder (Thermo Fisher Scientific, Waltham, MA, USA). Densitometry results represent the mean of three independent experiments (**B**); * *p* < 0.05, OI cells vs. normal cells; † *p* < 0.05, OI treated cells vs. OI untreated cells.

By using zymography assay, the presence of proenzymes (pro-MMP-2 and pro-MMP-9) and their active forms (MMP-2 and MMP-9), as well as MMP-1, was demonstrated (Figure 9A). Densitometric analysis of individual active forms of enzymes in OI showed similar MMP-1 and higher activities of MMP-2 and MMP-9, compared to those in normal cells (Figure 9B). MMP-2 activity was inhibited by 10 µM RA, 1 and 10 µg/mL RE, and LBE at all concentrations (0.1, 1, and 10 µg/mL). MMP-9 activity was inhibited by RA and LBE at all used concentrations and by RE at concentrations of 1 and 10 µg/mL. In turn, collagenase I (MMP-1) was only inhibited by the extracts at their higher concentrations (1 and 10 µg/mL).

## 4. Discussion

OI type I is the mildest type of the disease caused by stop or frameshift as well as splicing site mutations in *COL1A1* gene resulting in the haploinsufficiency of the α1 chain of collagen type I [43,44,45]. These mutations are known as quantitative in contrary to those which alter the amino acid sequence of type I collagen α chains and are called structural mutations [24,25,26,27]. The most common structural mutations are glycine substitutions in the triple helical domain of the α1 or α2 chains, which give the wide range of phenotypic severity from mild to lethal [23,24,25,26,27,28,29]. The quantitative and structural mutations affecting collagen type I have consequences in all tissues that produce this protein, however, the easy to obtain patients’ skin fibroblasts are the cell type commonly used for molecular testing. Apart from the few reports concerning structural mutations, where the expression and thermostability of mutant collagen type I in osteoblasts was slightly different than that of the skin fibroblasts [46,47], most collagen type I defects are similarly expressed and give symptoms not only in the bone but also in other connective tissues rich in this protein.

The greatest amount of collagen type I is found in skin, tendons, ligaments, bones, teeth, and the cornea [30]. Clinical symptoms commonly observed in OI patients include, in addition to abnormal bone formation and fragility, growth deficiency, blue or gray sclera, hearing loss, joint laxity, muscle weakness, dentinogenesis imperfecta, valvular regurgitation, and impaired pulmonary function [25,26,27,28,29]. In the skin collagen type I accounts for about 85–90% of the total collagen, the rest is collagen type III accounting for 10–15% and small amounts of dermal collagen are types IV, V, VI, and VII [30,31]. Collagen type I provides the dermis its structural integrity and has a significant impact on the properties of the skin because it is essentially responsible for the tensile strength and its mechanical properties, and it maintains skin firmness and elasticity. ECM of the skin is not only a structural but also a dynamic structure as collagen type I is a signaling molecule, which transmits the signals to the cell via integrins influencing the shape and behavior of cells [48]. In order to properly perform these functions, it is important to maintain collagen homeostasis. For example, abnormal collagen homeostasis associated with decreased biosynthesis of collagen under the influence of environmental factors, mainly UV radiation, is manifested by thin, fragile skin, which impairs its structural integrity and mechanical properties, and contributes to various skin diseases [31,49]. Abnormal skin structure and function in OI include thinness, translucency, bruising, decreased elasticity, and elastosis perforans serpiginosa [28,29]. Despite the growing research and knowledge of the genetic and molecular mechanisms of collagen biosynthesis and its abnormalities in this complex heterogeneous disease, OI is still an incurable disease. Therefore, research in this area is important to provide patients with the best possible treatments. There are many reviews on the pre-clinical treatment strategies, but existing therapies for OI are mainly based on an intravenous administration of bisphosphonates, however, with undesirable side effects [25,26,27,50,51,52]. Long-term inhibition of osteoclasts by bisphosphonates may reduce bone quality, leading to non-dynamic bone in which microdamages are not repaired and accumulate, likely causing an overall increase in bone fragility [25,26]. Unusual femoral fractures and delayed teeth eruption have been reported in children after long-term treatment with pamidronate [50].

Other antiresorptive therapies using monoclonal antibodies, such as Denosumab, against receptor activator of nuclear factor kappa-B ligand, Romosozumab against sclerostin (negative regulator of bone formation) or Odanacatib against cathepsin K (involved in the degradation of type I collagen) are currently under investigation in OI [50,51,52]. To improve patients’ condition growth hormone, growth factors (e.g., transforming growth factor), vitamin D supplementation are used [25,26,27,50,51].

Our study provides for the first time evidence of the beneficial effects of RA and extracts rich in this compound (RE and LBE) that either significantly reduced the quantitative defect of type I collagen in OI type I skin fibroblasts or, depending on their concentration, normalized to the level found in normal cells. After testing the cell responses to doses of the extracts and RA in the 1000–fold (0.1–100 µg/mL and 0.1–100 µM) range, an optimal stimulatory effect on collagen type I in relatively low concentrations of RA (0.1–10 µM RA) and extracts (0.1–10 µg/mL) was found. The reduction of type I collagen by about a half as a result of loss-of-function mutation in *COL1A1* gene and the increase under the influence of RA and the tested extracts (RE and LBE) were confirmed by assays at the mRNA (qRT-PCR) and protein (ELISA, SDS-urea PAGE, Western Blot) levels. At mRNA level, LBE showed about three-fold greater stimulating effect on the *COL1A1* gene as compared to RE. Taking into account the approximately three times higher concentration of RA in LBE (80.26 ± 1.24 mg/g) compared to RE (27.23 ± 0.54 mg/g) (Table 3), it can be hypothesized that RA as the main component of both extracts may be responsible for their beneficial effect on collagen type I. RA administration itself showed significant increase in collagen type I mRNA expression, the highest at a concentration of 1 µM, which corresponds to its content of approximately 4 µg/mL in LBE and 12 µg/mL in RE that at these concentrations significantly increased collagen type I expression. The quantification of procollagen type I with Elisa also showed the most effective stimulating effect by LBE as compared to RA and RE; in this case the level of procollagen in OI cells was comparable to the normal cells. Higher stimulating potential of LBE than RE at concentrations containing a comparable amount of RA may suggest that other compounds present in the extracts may be responsible for this effect. As shown in the Table 2, they contain many biologically active substances, some of them are common and others specific for one extract. For example, apigenin has only been identified in RE, whereas its glucoside (apigenin 7-*O*-glucoside) has been identified in LBE. Our previous studies have shown that apigenin had inhibitory while apigenin 7-*O*-glucoside slightly stimulatory effects on collagen biosynthesis in OI cells [21]. Based on these data, it can be assumed that the higher LBE stimulating effect on collagen type I may be the result of the synergistic action of RA and the apigenin 7-*O*-glucoside, although a different type of interaction between these and other compounds cannot be ruled out. A common component of both extracts is luteolin 7-*O*-glucoside (Table 2), and although in our research it was not quantified, other studies suggest significant differences in the content of it in RE and LBE [53,54]. In unpublished studies, we noted the stimulating effect of this flavonoid on type I collagen in normal human skin fibroblasts. If luteolin 7-*O*-glucoside shows a similar effect on OI cells with collagen deficiency, it can contribute to the action of rosemary and lemon balm extracts, but possible to a different degree due to the large differences in the concentration of this flavonoid in both extracts, given by these authors [53,54].

It should also be emphasized that, unlike drugs, the use of which usually involves more or less serious side effects, the use of these compounds of plant origin with many valuable properties, especially antioxidant, may bring a number of additional beneficial effects on the skin. Both RA and the extracts tested by us, at similar low concentrations, at which we revealed the stimulation of collagen type I biosynthesis, have been shown to protect against the effects of free radicals on skin fibroblasts. RA at the concentrations of 0.5 and 1.0 µM had ability to support the long-term lifelong growth of fibroblasts and increased tolerance to cellular stress by reducing the rate of telomere loss and reducing epigenetic molecular markers of cellular aging: 5-methyl-cytosine, 5-hydroxymethylcytosine, and oxidative stress marker 8-hydroxy-2′-deoxyguanosine [55].

Numerous reports indicate that RA has potential as a therapeutic agent in skin diseases caused by reactive oxygen species induced, among others, by solar UV radiation. UV is a very important environmental factor associated with an increased risk of many skin diseases including atrophy and neoplastic changes, and premature aging. It is responsible for many harmful symptoms, including inflammation, wrinkles and sagging skin, discoloration, erythema, swelling, immunosuppression, and hyperplasia [56]. RA at the concentration of 2.5 µM showed a protective effect on skin cells against UV radiation through the activation of the antioxidant enzymes (superoxide dismutase, catalase) and transcription factor (erythroid-derived 2)-like 2) [57]. The results of Rodríguez-Luna [58] suggest that RA at the concentration of 5 µM in combination with 5 µM fucoxanthin could be used as an interesting strategy in the counteracting oxidative damage in the skin induced by UVB. There is also a report of a protective effect of RA against the harmful effects of UVA on the formation of collagen type I fibers in normal human skin fibroblasts, however at a much higher concentration (62.5 µg/mL, which is equivalent to 174.6 µM) [59]. We had previously discovered the protective effect of RA (100–150 µM) against the negative influence of parabens (methyl- and propylparabens) on the skin collagen type I and III and the viability of human skin fibroblasts [22]. Most people are exposed to them throughout their lives due to their widespread presence in cosmetic and personal care products applied daily to the skin, and their excessive use can create a real health risk due to their estrogenic properties [60]. RA shows promise in the treatment of various inflammatory skin diseases such as psoriasis [61] and atopic dermatitis [12] as shown in in vitro and in vivo studies. It has been shown that RA and extracts rich in this compound exhibit anti-aged effects on the skin [13,14,15,16,17,18,62,63]. Rosemary rich in active antioxidants, microelements, and nutrients, exhibits protective potential against free radical damage and is essential in improving the quality of the skin. An interesting feature of the lemon balm extract is its melanogenic properties, which can also be used to protect the skin against UV radiation [17].

Skin homeostasis is mediated by the coordinated secretion by fibroblasts of MMPs involved in the degradation of the ECM components, including collagen [49]. Their secretion is stimulated by oxidative stress, UV radiation, and cytokines and they play an important role in in wound healing, skeletal growth, and remodeling as well as various pathophysiological processes, such as photoaging, inflammation, angiogenesis, and cancer [49]. In skin fibroblasts, MMPs are secreted in a latent form and the active forms appear in the medium after culturing for 72–96 h [64]. In our study to test the activity of MMP-1, -2, and -9 after 24 h of cell culture, MMPs were activated with APMA. Depending on the concentrations of RA and extracts, they did not have an effect or showed inhibitory effect, which may be beneficial in many processes including collagen degradation, which they mediate.

In summary, both RA and extracts (RE and LBE) at low concentrations non-toxic to the fibroblasts showed the beneficial effects on type I collagen and MMPs (MMP-1, -2, and -9) involved in collagen and skin homeostasis. Extracts, especially LBE, turned out to be more effective than RA itself in stimulation of the expression of type I collagen at the protein level. It is important to note that the extracts are generally cheaper and more convenient to produce for the use as nutraceuticals. In an in vivo study involving eleven healthy young individuals, it was shown that the intake of a single dose of LBE extract containing 500 mg of RA per day was safe [65]. The goal of this study was to use the extract to treat age-related neurodegenerative disorders; however, long-term safety studies in this population are needed to confirm these results. The stronger effect of the extracts on type I collagen compared to RA demonstrated in our study requires further research to identify compounds other than RA responsible for their more effective action. Despite the identification of a large amount of polyphenolic compounds with broad biological activity, there are little, with the exception of our reports [20,21,22], research on their influence on this key protein of the human body.

Of note, in data interpretation it is important to take into consideration the following limits of the present study. First, our data are limited to the use of a single OI skin fibroblast line and the test on more OI type I patients cells will be necessary to strengthen the results. Secondly, considering the pharmacokinetics of RA or other components of the extracts (their limited oral absorption or rapid metabolism), there may be large differences between the results obtained from our in vitro study versus in vivo administration. At the target site of action in the body, it is possible that concentrations may be significantly lower than the ones used. However, the concentrations of the tested extracts and RA at which the optimal effect on collagen was obtained in our study can be considered similar or much lower compared to those used in many other studies. Finally, additional data on their pharmacokinetics are necessary in order to use them in the co-therapy of this disease or also other disorders associated to collagen type I deficiency.

## 5. Conclusions

This study demonstrates new clinically relevant properties of RA and of RA containing extracts (RE and LBE) related to their potential to promote the expression of type I collagen in the skin fibroblasts of type I OI patient. Noteworthy, their lack of toxicity up to a concentration of 100 µM RA and 100 µg/mL of extracts, and their beneficial effect on collagen type I at low concentrations, as well as a number of their other valuable health promoting effects, are of particular relevance for therapy. RA may play a major role in the action of RE and LBE on collagen type I; however, testing the action of other components in future research may better elucidate the mechanism of action of these extracts.

## Figures and Tables

**Table 1:** Regression data, limit of detection (LOD) and limit of quantification (LOQ), accuracy and precision obtained during LC-PDA-MS method optimization.

Analyte	Regression Equation ^a^	R^2^	Linear Range [mg/mL]	LOD [mg/mL]	LOQ [mg/mL]	Accuracy [%]	Precision [%]
RA	y = 4338.7x + 85.712	0.9998	0.1–3.0	0.0694	0.2103	99.88 ± 2.53	1.73

^a^—the value for y corresponds to the peak area and x to the concentration, respectively.

**Table 2:** Qualitative analysis of rosemary extract (RE) and lemon balm extract (LBE) by liquid chromatography-photodiode array detection-mass spectrometry (LC-PDA-MS).

No	Rt (min)	Tentatively Identified Compounds	λ Max (nm)	[M + H]^+^/[M + H] (*m*/*z*)	Extracts
1	1.50	Quinic acid ^a^	290	-/191	RE, LBE
2	9.88	2-hydroxy-3-(3,4-dihydroxyphenyl)-propanoic acid ^b^	283	-/197	RE, LBE
3	17.54	Medioresinol ^c^	290	-/387	RE
4	20.20	Gallocatechin ^d^	280	-/305, 225	RE
5	20.60	Hydroxyjasmonic acid sulphated ^b^	275, 325	-/305, 225	LBE
6	21.20	Salvianolic acid derivative isomer 1 ^b^	275, 325	-/537, 493, 295	LBE
7	22.68	Luteolin glucosylrhamnoside ^b^	280, 330	-/593	LBE
8	23.05	Luteolin 7-*O*-glucoside ^s^	282, 333	287, 449/285, 447	RE, LBE
9	23.88	Nepetin 7-*O*-glucoside ^a^	272, 345	317, 479/477	RE
10	24.71	Apigenin 7-*O*-glucoside ^s^	280, 338	271, 433/-	LBE
11	24.94	Hesperitin-7-rhamnoglucoside ^s^	284, 330	303, 610/301, 609	RE
12	25.12	Salvianolic acid B ^b^	280, 325	-/717	LBE
13	25.40	Hispidulin 7-*O*-glucoside ^s^	274, 334	301, 463/461	RE
14	25.81	Rosmarinic acid ^s^	290, 330	361, 343, 505/359	RE, LBE
15	26.12	Sagerinic acid ^b^	280, 330	-/719, 359, 161	LBE
16	26.15	Luteolin 7-*O*-glucuronide ^b^	268, 340	287, 463/461	RE, LBE
17	26.45	Nepetin derivative ^a^	275, 335	317, 685/683	RE
18	27.12	Cirsimaritin derivative ^a^	278, 332	315, 477, 655/653	RE
19	27.12	Lithospermic acid isomer 1 ^b^	290, 325	-/537, 493, 359	LBE
20	27.32	Luteolin 3′-O-(O-acetyl)-glucuronide isomer 1 ^a^	268, 335	505/285, 459, 503	RE
21	27.71	Lithospermic acid isomer 2 ^b^	290, 325	-/537, 493, 359	LBE
22	27.77	Luteolin 3′-O-(O-acetyl)-glucuronide isomer 2 ^a^	268, 335	505/285, 459, 503	RE
23	28.14	Luteolin 3′-O-(O-acetyl)-glucuronide isomer 3 ^a^	268, 335	505/285, 459, 503	RE
24	28.32	Rosmarinic acid sulphated isomer ^b^	290, 328	-/439, 359	LBE
25	29.90	Apigenin ^s^	268, 335	271/269	RE
26	30.15	Salvianolic acid derivative isomer 2 ^b^	290, 327	-/715	LBE
27	30.50	Hispidulin ^s^	276, 334	301/299	RE
28	30.82	Salvianolic acid derivative isomer 3 ^b^	290, 327	-/715	LBE
29	32.04	Rosmanol isomer 1 ^a^	284	369/283, 301, 345	RE
30	32.26	Cirsimaritin ^s^	275, 335	315/313	RE
31	32.80	Rosmanol isomer 2 ^a^	284	369/283, 301, 345	RE
32	33.65	Rosmanol isomer 3 ^a^	288	369/283, 301, 345	RE
33	34.00	Genkwanin ^a^	268, 334	285/283	RE
34	38.38	Miltipolone isomer ^a^	285	345/299	RE

Comparison: ^s^—reference substance, ^a^—Borrás Linares et al. [39]; ^b^—Ozarowski et al. [40]; ^c^—Hossain et al. [41]; ^d^—Nie et al. [42].

**Table 3:** Concentration of rosmarinic acid (RA) in rosemary extract (RE) and lemon balm extract (LBE).

Sample	RA Concentration [mg/g]	SD [mg/g]	RSD [%]
RE	27.23	0.54	2.01
LBE	80.26	1.21	1.51

SD—standard deviation, RSD—relative standard deviation.

## Data Availability

Data is contained within the article.

## Abbreviations

APMA	4-aminophenylmercuric acetate
BSA	Bovine serum albumin
DMEM	Dulbecco’s minimal essential medium
DMSO	Dimethyl sulfoxide
ECM	Extracellular matrix
FA	Formic acid
FBS	Fetal bovine serum
LBE	Lemon balm extract
LC-PDA-MS	Liquid chromatography-photodiode array detection-mass spectrometry
MMPs	Matrix Metalloproteinases
MTT	(4,5-dimethylthiazol-2-yl)-2,5-diphenylte-trazolium bromide
OI	Osteogenesis imperfecta
PBS	Phosphate-buffered saline
PDA	Photo-diode array
qRT-PCR	Quantitative Real-time PCR
RA	Rosmarinic acid
RE	Rosemary extract
RIPA	Radioimmunoprecipitation assay buffer
SD	Standard deviation
SDS	Sodium dodecyl sulfate
SDS-urea PAGE	SDS-urea polyacrylamide gel electrophoresis
UV	Ultraviolet
UPW	Ultra pure water
